# Biomarker discovery in galactosemia: Metabolomics with UPLC/HRMS in dried blood spots

**DOI:** 10.3389/fmolb.2023.1154149

**Published:** 2023-04-04

**Authors:** Ahmad N. Alodaib, Refat M. Nimer, Rowan Alhumaidy, Alaa Alhenaky, Mai Abdel Jabar, Reem H. AlMalki, Anas M. Abdel Rahman

**Affiliations:** ^1^ Metabolomics Section, Department of Clinical Genomics, Center for Genomics Medicine, King Faisal Specialist Hospital and Research Centre (KFSHRC), Riyadh, Saudi Arabia; ^2^ Department of Biochemistry and Molecular Medicine, College of Medicine, Al Faisal University, Riyadh, Saudi Arabia; ^3^ Department of Medical Laboratory Sciences, Jordan University of Science and Technology, Irbid, Jordan; ^4^ Department of Botany and Microbiology, College of Science, King Saud University, Riyadh, Saudi Arabia

**Keywords:** biomarker, dried blood spot, galactosemia, metabolomics, newborn screening (NBS), ultra-performance liquid chromatography

## Abstract

**Introduction:**Galactosemia (GAL) is a genetic disorder that results in disturbances in galactose metabolism and can lead to life-threatening complications. However, the underlying pathophysiology of long-term complications in GAL remains poorly understood.

**Methods:** In this study, a metabolomics approach using ultra-performance liquid chromatography coupled with high-resolution mass spectrometry was used to investigate metabolomic changes in dried blood spots of 15 patients with GAL and 39 healthy individuals.

**Results:** The study found that 2,819 metabolites underwent significant changes in patients with GAL compared to the control group. 480 human endogenous metabolites were identified, of which 209 and 271 were upregulated and downregulated, respectively. PA (8:0/LTE4) and ganglioside GT1c (d18:0/20:0) metabolites showed the most significant difference between GAL and the healthy group, with an area under the curve of 1 and 0.995, respectively. Additionally, the study identified potential biomarkers for GAL, such as 17-alpha-estradiol-3-glucuronide and 16-alpha-hydroxy DHEA 3-sulfatediphosphate.

**Conclusion:** This metabolomics study deepened the understanding of the pathophysiology of GAL and presented potential biomarkers that might serve as prognostic biomarkers to monitor the progression or support the clinical diagnosis of GAL.

## 1 Introduction

Galactosemia is an autosomal recessive disorder caused by a defect in the enzyme galactose-1-phosphate uridyltransferase (GALT) (Isselbacher et al., 1956; Berry, 2015). This enzyme is needed to convert galactose (a sugar found in dairy, fruit, and some foods) into the body’s primary energy source (Leloir, 1951). If this enzyme is deficient, galactose accumulates to toxic levels in the body, leading to a severe neurological and metabolic disorder. Symptoms of galactosemia can include vomiting, lethargy, seizures, enlarged liver, jaundice, kidney failure, and changes in brain development.

A defective gene causes the classic form of GAL on chromosome 3. It is characterized by severe deficiency of all three enzymes required for the metabolism of galactose to glucose: galactose-galactokinase (GALK) (GAL type II), galactose-1-phosphate uridylyltransferase (also known as GALT), and UDP-galactose-4-epimerase (GALE) enzymes (GAL type III) (Pasquali et al., 2018). Without these enzymes, galactose is not converted to glucose, accumulates in the body, or is excreted in the urine. The incomplete breakdown of galactose then affects numerous metabolic pathways, which results in several serious symptoms and potential long-term complications such as cataracts, liver damage, and an increased risk of developing neurological disorders. The primary treatment for galactosemia is a strict low-galactose diet. All sources of galactose and lactose, such as cow’s milk, need to be eliminated from the diet. An alternative lactose-free, low-galactose breast milk or formula can be used for nutrition. Depending on the individual, the diet may need to be modified periodically. Infants with galactosemia should have regular check-ups with their doctor to ensure proper nutrition, growth, and development.

The Leloir pathway is the metabolic pathway used to convert galactose into glucose. It begins with the conversion of galactose to glucose-1-phosphate, which is catalyzed by the enzyme galactokinase. Glucose-1-phosphate is then converted to glucose-6-phosphate by uridyl transferase and, finally, glucose by the enzyme glucokinase. At the same time, galactose is rapidly metabolized *via* the Leloir pathway once it enters the cell, where initially, GALK catalyzes the phosphorylation of galactose (Walter and Fridovich-Keil, 2019). Then GAL transforms UDP-glucose and galactose 1-phosphate into glucose-1-phosphate and UDP-galactose. Finally, GALE catalyzes the conversion of UDP-galactose to UDP-glucose (Timson, 2016). Additionally, type IV galactosemia is a newly found hereditary metabolic disorder. It is caused by mutations in the galactose mutarotase gene, which results in the diminished activity of the enzyme galactose mutarotase. This enzyme catalyzes the interconversion of the α- and β-anomers of d-galactose and several other monosaccharides (Banford and Timson, 2021). However, the mechanisms of GAL disorder are still poorly understood (van Weeghel et al., 2018). The clinical complications associated with classical galactosemia include cataracts, developmental delays, learning disabilities, speech problems, failure to thrive, intestinal problems, and liver damage. If left untreated, galactosemia can lead to life-threatening conditions such as sepsis, multiple organ failure, and death. It is also associated with an increased risk of ovarian failure in females (Succoio et al., 2022).

The pathophysiology of the long-term complication in GAL needs to be better understood, and predictive biomarkers need to be included (Hermans et al., 2022). Prognostic uncertainty may lead to unnecessary or harmful treatment for patients with GAL, which burden patients and parents (Knerr et al., 2015). Even though neonatal detection and dietary restriction of galactose may change the clinical picture of a newborn, it does not stop long-term problems from happening (Succoio et al., 2022).

Most of the world’s newborn screening (NBS) programs include a screen for GAL; despite the level of its false discovery rate, even at early diagnosis, there are often long-term complications (Ohlsson et al., 2011). Fanconi-Bickel disease, liver illness, glycogen storage disease type XI, and even certain drugs may all cause false-positive screening findings in infants (Peduto et al., 2004; Kotb et al., 2019). Most NBS programs depend on measuring GAL activity in DBS to diagnose GAL.

Total galactose (galactose + galactose-1-phosphate) is measured in around 30% of NBS programs as a main screening approach or in conjunction with GAL testing in DBS. However, false negative screening results for GAL may be seen in babies who are given lactose-free formula or who are receiving complete parenteral nutrition if the diagnosis is based only on total galactose (Pasquali et al., 2018). A second analysis of dried blood from the same newborn screening card is undertaken to monitor particular metabolites or metabolic pathways to overcome the issue of non-specific first-line parameters and the resultant high false positive rate (Lehotay et al., 2011). Furthermore, positive results in screening tests, clinical examination, and biochemical and molecular diagnostics are required to confirm patients with GAL. However, based on the biochemical, enzymatic, and genetic information, that is, now available, it is not feasible to provide an accurate prediction of the clinical prognosis at the time of diagnosis.

Because there are no validated biomarkers for the diagnosis and prognosis of GAL, and no specific and reliable treatment regimens, more studies on GAL should be conducted. Metabolomics describes analyzing all metabolites (compounds with low molecular weight, generally 1,500 Da) present in a particular sample acquired from a biological system (Patti et al., 2012; Dahabiyeh et al., 2020; Gu et al., 2020). Metabolomics has emerged as a potentially useful diagnostic and prognostic tool that might explain disease pathogenesis (Masood et al., 2021; Jacob et al., 2022; Jans et al., 2022). There currently needs to be more investigations on the pathophysiology of GAL that concentrate on urine or blood metabolomics profiling (Taylor Fischer et al., 2019; Hermans et al., 2022). Therefore, investigating the GAL metabolomics profile may aid in finding potential biomarkers, shed light on the mechanisms behind the disease’s progression, and ultimately aid in its early detection.

In this study, metabolomics employing ultra-performance liquid chromatography coupled with high-resolution mass spectrometry (UPLC/HRMS) was used to detect and quantify differences in metabolite levels between GAL and healthy groups that could potentially serve as biomarkers for the diagnosis or monitoring of GAL and could also provide insights into the underlying biological mechanisms of the disorder.

## 2 Materials and methods

### 2.1 Characteristics of the study population

Fifty-four DBS samples were collected from genetically and biochemically confirmed GAL (*n* = 15) patients at King Faisal Specialist Hospital and Research center (KFSHRC) and healthy controls (*n* = 39). These healthy controls were age-gender matched with the patient group. 4 out of 19 GAL patients and 7 out of 46 healthy controls were excluded from this study due to 1) inability or unwillingness to provide informed consent or 2) diagnosis with conditions other than GAL. The Research Ethics committee approved this study and Institutional Review Board at KFSHRC (RAC# 2160027). It was performed following the ethical standards of the Declaration of Helsinki.

### 2.2 Metabolites extraction

The polar metabolites were extracted from DBS samples using our developed standard protocol (Jacob et al., 2018). Five 3 mm size DBS disks were used for metabolite extraction using methanol, acetonitrile, and water (40:40:20%) for protein precipitation. The mixture was mixed at 25°C and 600 rpm for 2 hours in a thermomixer (Eppendorf, Germany). Pooled QC samples were prepared using aliquots from the study samples. Afterward, the supernatants were transferred to another set of tubes, evaporated in SpeedVacc (Christ, City, Germany), and stored at −80°C until LC-MS analysis.

### 2.3 UPLC/HRMS

The metabolomics profile for the study samples was collected using our laboratory’s applicable standard protocol (Jaber et al., 2022). In detail, the dry extracted samples were resuspended with 50% mobile phase A and B (A: 0.1% formic acid in dH_2_O, and B: 0.1% formic acid in 50% MeOH and ACN). The extracted metabolites were chromatographed using an Acquity UPLC using XSelect HSS C18 (100 × 2.1 mm, 2.5 μm) column (Waters Ltd., Elstree, United Kingdom). A gradient mobile phase elution was scheduled in this method as follows: 0–16 min 95%–5% A, 16–19 min 5% A, 19–20 min 5%–95% A, and 20–22 min, 95%–95% A, all at a flow rate of 300 μl/min. The eluted molecules were detected using a Waters Acquity UPLC connected to a Waters Xevo G2-S QTOF high-resolution mass spectrometry system. In separate runs, the molecules were ionized using positive and negative electrospray ionization modes (ESI+, ESI-). In ESI+, the capillary voltage was set to 3.20 kV. The cone voltage was 40 V, the desolvation temperature was 500°C, the nitrogen desolvation gas flow to 800 L/h, and the cone gas flow was 50 L/h. In ESI−, a capillary voltage of −3 kV was used. The collision energies of low and high functions were set at 0 V and 10–50 V, respectively, in MS^E^ mode. The mass spectrometer was calibrated, as recommended by the vendor, with sodium formate in the range of 100–1,200 Da in both ionization modes. Accurate mass measurements were maintained by continuously infusing leucine-enkephaline lock mass compound (ESI + m/z 556.2771, ESI- m/z 554.2615) and alternating between the sample and the reference every 45 and 60 s for ESI+ and ESI-, respectively. The lock spray was 10 μl/min, 0.5 s scan time, cone voltage 30 V, collision energy 4 eV. The data-independent acquisition was performed in continuum mode with Masslynx™ V4.1 workstation (Waters Corporation, MA, United States).

### 2.4 Data processing and statistical analysis

Peak picking and alignment of detected ion (m/z, Rt) were processed using Progenesis QI v.3.0 software from Waters (Waters Corporation, MA, United States).

The raw data were deposited in MetaboLight (accession Number MTBLS6996).

Multivariate statistical analysis was performed using MetaboAnalyst v5.0 (McGill University, Montreal, QC, Canada) (Worley and Powers, 2013). Firstly, data were subjected to log transformation, mean centering, and Pareto scaling and then used to generate principal component analysis (PCA), partial least squares-discriminant analysis (PLS-DA), and orthogonal projections to latent structures discriminant analysis (OPLS-DA) models. OPLS-DA models were evaluated using the fitness-of-model (R2Y) and predictive ability (Q2) values.

Univariate analysis was performed using Mass Profiler Professional (MPP) Software (Agilent Technologies, Inc., Santa Clara, CA, United States). The total sample median was used to normalize the signal and ensure normal distribution. Volcano Plot analysis was performed to identify significantly alters between GAL patients and healthy control using Moderated T. Test, false discovery rate (FDR) corrected *p*-value ≤0.05 and fold change (FC) cut-off of 2. Venn diagrams were developed using MPP Software (Agilent Technologies, Inc., Santa Clara, CA, United States).

Pathway analysis and biomarkers linked with GAL disorder were performed using MetaboAnalyst v5.0 (McGill University, Montreal, QC, Canada)—a pathway view of statistically significant pathways flagged from the metabolome view based on matched metabolites. The pathways are arranged based on the *p*-value (y-axis), which indicates the pathway enrichment analysis, and pathway impact values (x-axis) representing pathway topology analysis. In addition, Receiver Operating Characteristic (ROC) curves were created using the PLS-DA approach in the MetaboAnalyst v 5.0 for global analysis to identify possible biomarkers. Metabolites were putatively identified based on the exact mass searched against different databases, including Human Metabolome Database and METLIN. The exogenous compounds, such as drugs, food additives, and environmental compounds, were excluded from the final list.

## 3 Results

### 3.1 Feature detection and metabolites identification

Using the UPLC/HRMS data, comprehensive untargeted metabolomics analyses were performed on the DBS samples obtained from 15 GAL patients and 39 healthy controls. In total, 25,607 m/z features were detected in positive (*n* = 12,541) and negative (*n* = 13,066) ionization modes. After applying the filter of 80% of all samples, 20,775 features remained to statistically evaluate among the patients with GAL and healthy control, as described in the method section.

### 3.2 Metabolomics profiling for GAL compared to control

**FIGURE 1:**
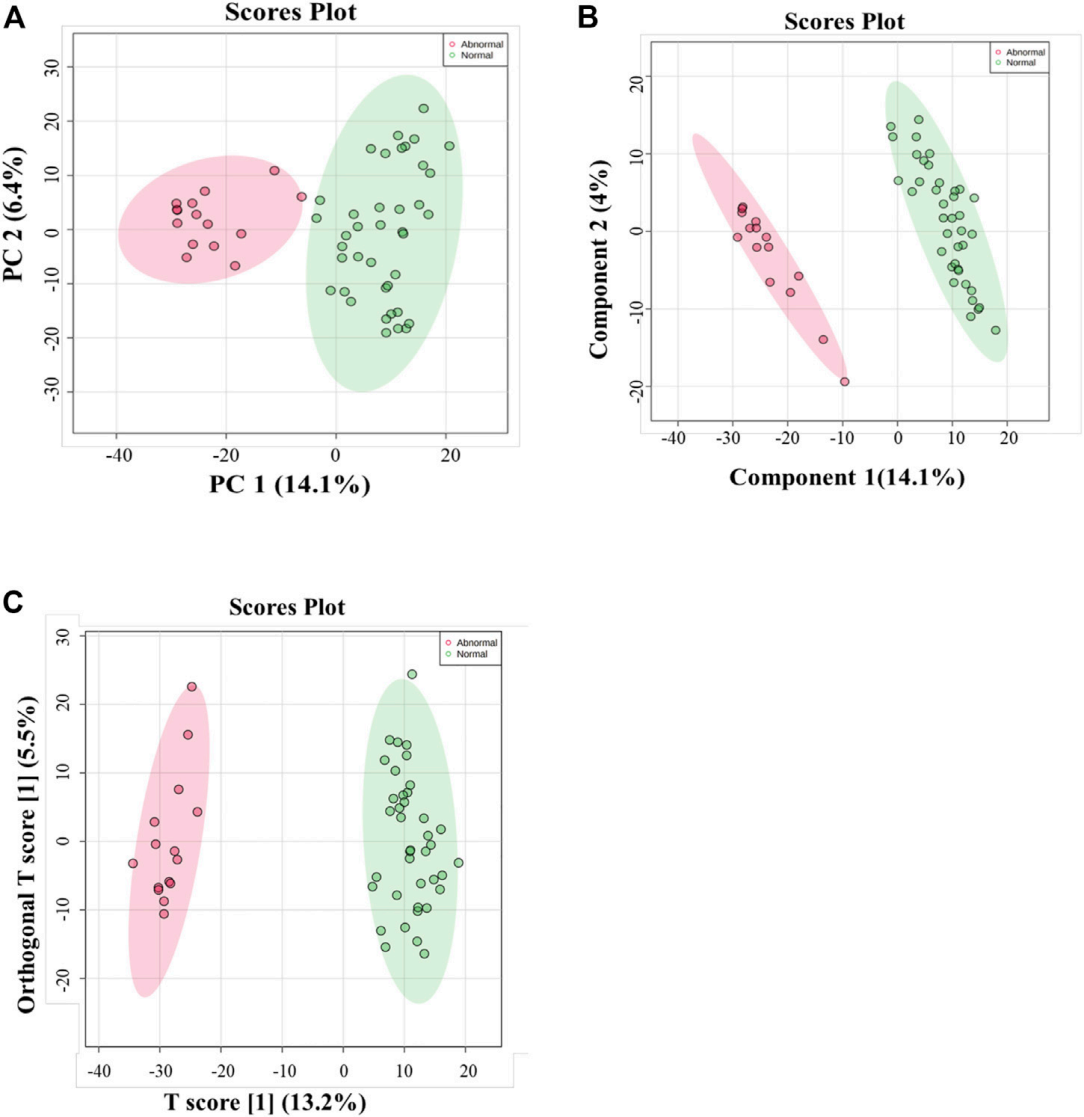
**(A)** Principal component analysis (PCA) model for 54 samples obtained from 15 GAL patients and 39 control that showed a clear separation between the two groups (GAL patients and healthy control). **(B)** PLS-DA Score Plots revealed a clear separation between the groups (GAL patients and healthy control). **(C)** Orthogonal partial least squares-discriminant analysis (OPLS-DA) score plot showed evident separation between two groups (GAL patients and healthy control). The robustness of the created models was evaluated by the fitness of the model (R2Y = 0.972) and predictive ability (Q2 = 0.856) values.

Multivariate and univariate analyses were used to determine whether metabolites were significantly different in GAL compared to the control group. As a result of unsupervised, PCA revealed good clustering between GAL patients (red) and the healthy group (green). The total variance of the first two principal components contributed 20.5% in the PCA model for the two study groups (PC 1 = 14.1% and PC 2 = 6.4%) (Figure 1A). Once separation had been assessed, PLS-DA and OPLS-DA were applied to maximize the separation of the groups observed by PCA. The scores plot from the PLS-DA (Figure 1B) and the OPLS-DA (Figure 1C) showed clear group separation, which validated the PCA results. The OPLS-DA model yielded satisfactory fitness of the model (R2Y = 0.972) and predictive ability (Q2 = 0.856) values (Figure 1C). The contributing metabolites in these models’ separation between study groups were explored using univariate analysis (Student T. Test and fold change analyses).

**FIGURE 2:**
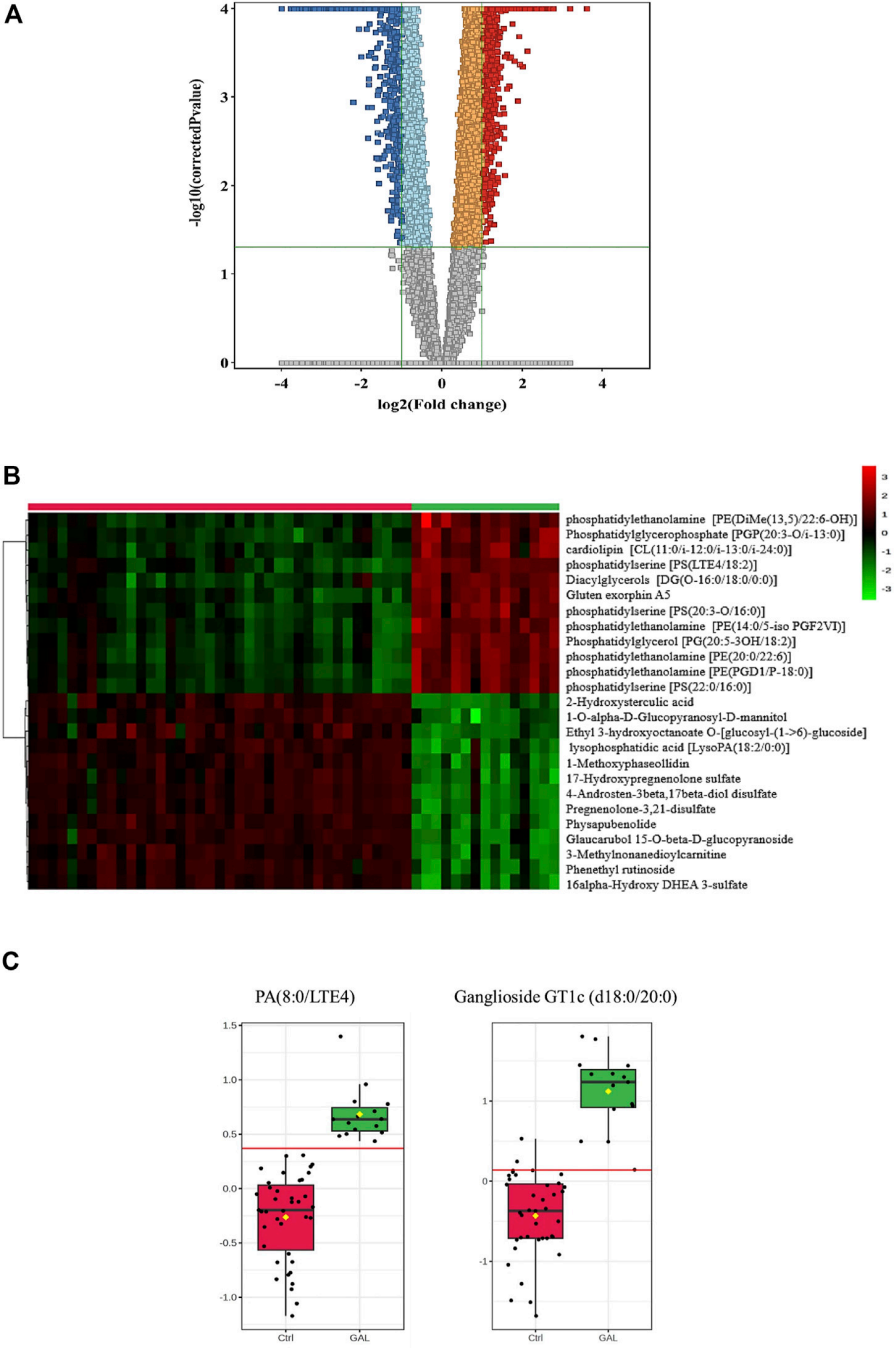
**(A)** Volcano Plot demonstrates the statistically significant altered metabolites filtered between the two groups (GAL patients and healthy control) that 2,819 significantly were dysregulated metabolites (FDR *p*-value ≤0.05, FC 2), of which 1,300 (red) and 1,519 (blue) metabolites were up-and downregulated in GAL patients compared to healthy control, respectively. Red and blue refer to up-and downregulated metabolites, respectively. Orange and light blue squares refer to metabolites that failed to pass fold change cutoffs and were up- and downregulated, respectively. Gray square metabolites failed to pass both cutoffs. **(B)** Heat map representing the top 25 significantly (*p* < 0.05) altered metabolites between the two study groups; healthy control (red) and GAL patients (green). **(C)** Boxplots for a couple of metabolites [Ganglioside GT1c (d18:0/20:0) and PA (8:0/LTE4)] where green represents GAL patients and red represents Control.

Next, a binary comparison between the GAL group and healthy control using volcano plot analysis revealed that 1,300 metabolites were upregulated (red) whereas 1,519 metabolites were downregulated (blue) in GAL patients compared to healthy control (FDR *p* ≤ 0.05, FC cut-off of 2), respectively (Figure 2A). Four hundred eighty metabolites were annotated as endogenous human metabolites and are listed in Supplementary Table 1. Further examination using hierarchical clustering analysis (HCA) in Figure 2B depicts differences in the abundance of the top 25 perturbed metabolites between GAL and control groups.

**FIGURE 3:**
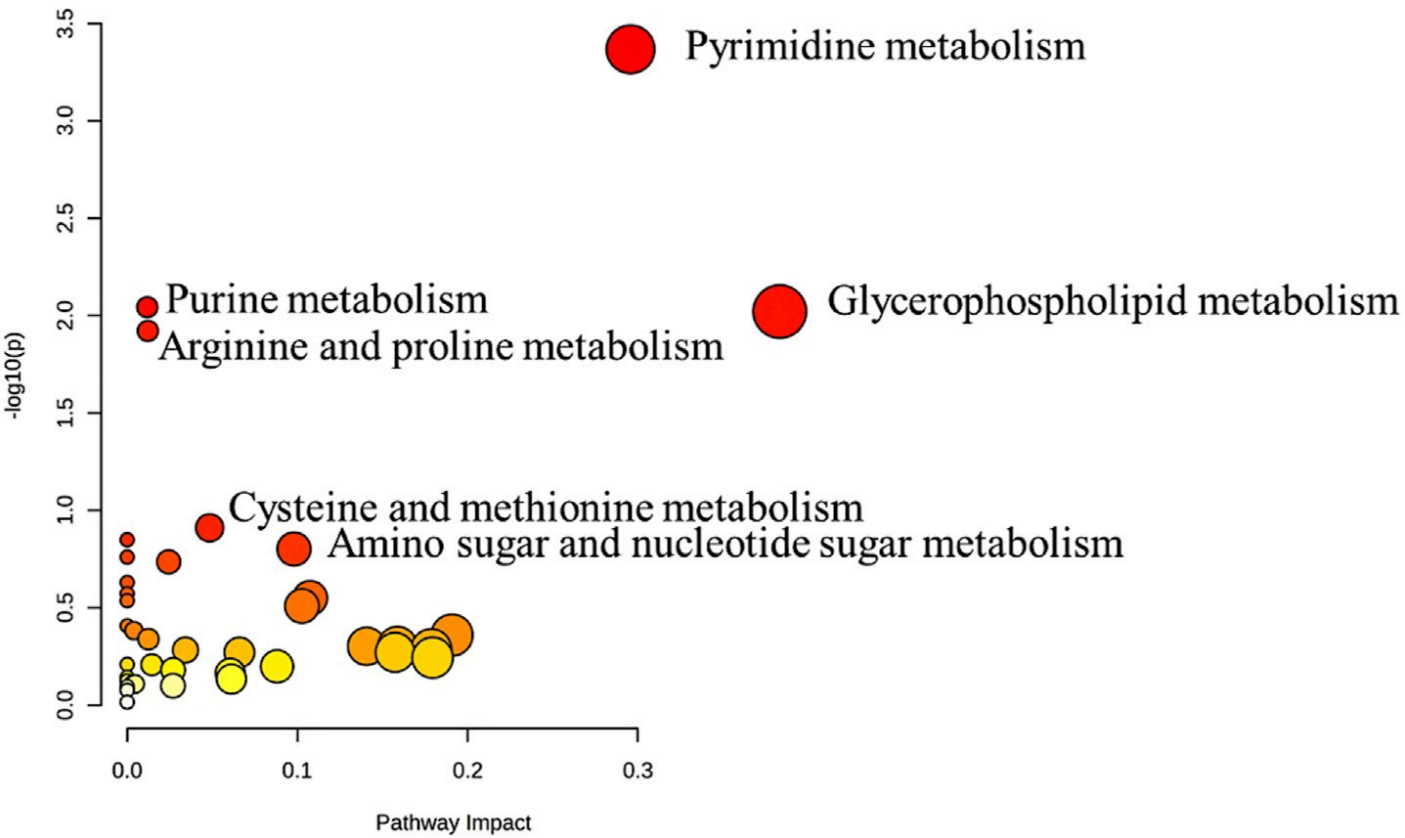
Pathway Analysis for the significant metabolites dysregulated between GAL patients and healthy control. Four hundred eighty metabolites were ultimately identified as human endogenous metabolites, where 209 and 271 were up and downregulated, respectively. Colors (varying from yellow to red) mean the metabolites are in the data with different significance levels (*p*-value).

The metabolic pathway analysis revealed that pyrimidine metabolism is the most significantly altered pathway in GAL compared to the control, as displayed in Figure 3.

**FIGURE 4:**
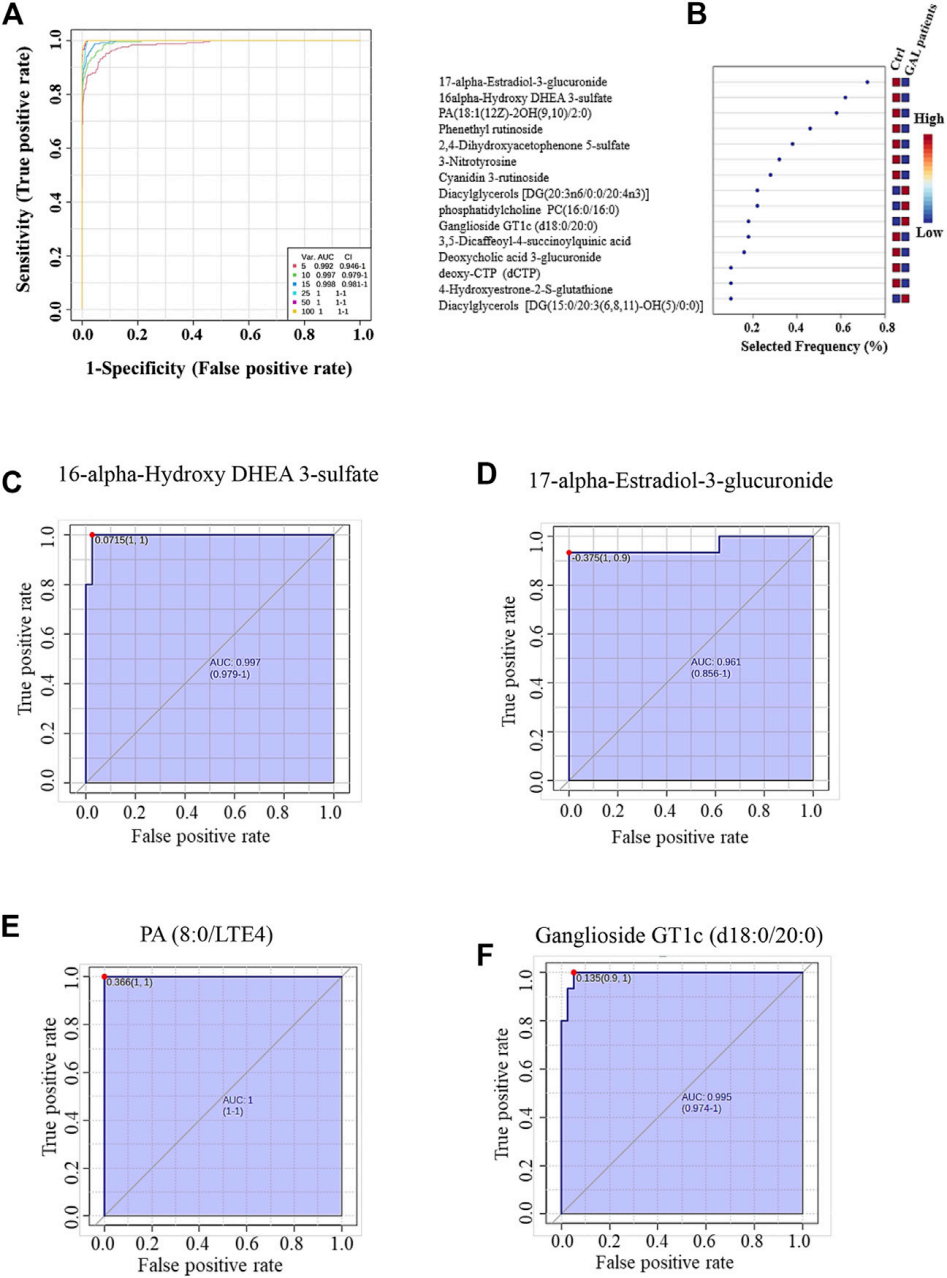
**(A)** The Receiver Operating Characteristics (ROC) curve was generated by the OPLS-DA model, with Area Under the Curve (AUC) values calculated from the combination of 5, 10, 15, 25, 50, and 100 metabolites. **(B)** The frequency plot shows the significantly dysregulated endogenous metabolites between the study groups. Representatives downregulated metabolites with their ROC curves are demonstrated for 16-alpha-hydroxy DHEA 3-sulfatediphosphate (AUC (0.997) **(C)** and 17-alpha-estradiol-3-glucuronide AUC (0.961) **(D)** in GAL patients. Furthermore, PA (8:0/LTE4) AUC (1) **(E)** and ganglioside GT1c (d18:0/20:0) AUC (0.995) **(F)** as examples of upregulated metabolites in GAL patients.

The ROC analysis was used to identify metabolites that might act as potential biomarkers and to assess their diagnostic accuracy (Figure 4). Multivariate exploratory ROC analysis was created using PLS-DA for classification and feature ranking. Figure 4A shows that the top-ranked metabolites in ROC curves show the area under the curves (AUCs) ranging from 0.992 to 1; confidence Interval (CI): 0.946–1 and 1-1. The selected frequency plots represent the significant features of the expressed metabolites in the patients with GAL and control groups (Figure 4B). The selected frequency plot shows metabolites, such as 16-alpha-hydroxy DHEA 3-sulfatediphosphate (UDP) and 17-alpha-estradiol-3-glucuronide to be downregulated in patients with GAL in comparison to the control group with AUC 0.997 and 0.961, respectively (Figures 4C, D). In comparison, metabolites such as phosphatidylcholine and diacylglycerols (DG) (20:3n6/0:0/20:4n3) were upregulated. Moreover, phosphatidic acid (PA (8:0/LTE4)) (Figure 4E) and ganglioside GT1c (d18:0/20:0) (Figure 4F) were upregulated in patients with GAL compared to the control group with an AUC 1 and 0.995, respectively shows highest discriminatory power.

## 4 Discussion

The urgent need for novel biomarkers for early diagnosis and prognosis prediction of GAL disorder has prompted the investigation of potential biomarkers using various experimental approaches. This study conducted metabolomics analyses to identify biomarkers with UPLC/HRMS by following changes in the metabolic profiles of patients with GAL. As an autosomal recessive hereditary genetic disorder, GAL can result in life-threatening health complications unless lactose is eliminated from the diet immediately after birth (Berry, 2021). The clinical outcome of patients with GAL varies widely (Welsink-Karssies et al., 2020). Additionally, pitfalls in diagnosing GAL are present due to false negative and positive newborn screening results (Pasquali et al., 2018). Thus, there is an urgent need to find novel biomarkers for early diagnosis and prognosis prediction of GAL disorder.

Despite the emerging field of metabolomics as the newest Omics platform that focuses on metabolites, small molecules (<1,500 Da) hold promise to shine a light on the molecular mechanisms of several diseases, which may help for diagnostic and therapeutic purposes (Jacob et al., 2019), very few studies have focused on GAL in humans biological fluids (Janeckova et al., 2015; Taylor Fischer et al., 2019; Hermans et al., 2022).

A volcano plot analysis was utilized to identify potential biomarkers of GAL. 2,819 metabolites showed significant differences between the GAL group and the control group. In the heatmap, the top 25 metabolites with the most significant differences in abundance between the groups were visualized and identified as potential biomarkers for GAL. Among these metabolites, phosphatidylethanolamine, which is a category of phospholipids present in biological membranes, was found to be the most significantly upregulated metabolite in the GAL group, confirming the increase of phosphatidylethanolamine in complications of GAL such as neurological impairments and cataracts (Jernigan Jr et al., 2005; López de Frutos et al., 2022).

Pyrimidine metabolism was the most significant pathway that significantly altered between GAL and healthy controls. The pyrimidines are the building blocks of DNA and RNA. They also form active intermediates in carbohydrate metabolism, such as UDP-glucose (Dewulf et al., 2021). Furthermore, UDP-glucose is an organic pyrimidine nucleotide sugar molecule (Ng et al., 2015). In the physiological process, the UDP-glucose is transformed into UDP-galactose in the presence of GAL (Veiga-da-Cunha et al., 2020). While in patients with GAL, the activity of the GAL enzyme is absent or barely detectable (Berry, 2021). Thus, a close link between GAL and alteration in pyrimidine metabolism was shown previously (Taylor Fischer et al., 2019), which matched our result.

We found that ganglioside GT1c and PA (8:0/LTE4) showed the highest discrimination ability between GAL and the control group. Ganglioside GT1c is a glycosphingolipid (ceramide and oligosaccharide) with one or more sialic acids widely distributed throughout the body, especially abundant in the brain and other parts of the central nervous system (Vukelic et al., 2001). Galactosylation of complex molecules and the production of various glycoproteins/glycolipids rely on GALE, which is also responsible for the interconversion of UDP-N-acetylglucosamine (UDP-GlcNAc) and UDP-N-acetylgalactosamine (UDP-GalNAc) (Banford et al., 2021). Therefore, neurological complications in patients with GAL may result from prenatal-neonatal toxicity or persistent glycoprotein and glycolipid synthesis abnormalities (Coman et al., 2010).

Moreover, oxidized phosphatidic acid is known as PA (8:0/LTE4), which is upregulated in our present study. A phosphate moiety occupies a glycerol substitution site in oxidized phosphatidic acids, which are glycerophospholipids in which at least one of the fatty acyl chains has undergone oxidation (Wishart et al., 2022)—metabolic perturbations in glycerophospholipids found in patients with GAL (Taylor Fischer et al., 2019).

One of the complications of GAL is liver cirrhosis since GAL is a common metabolic liver disorder of childhood (Sahoo et al., 2015). In the liver, glucuronidation takes place, where it is used to assist in the excretion of toxic substances, drugs, or other substances by attaching glucuronic acid *via* a glycosidic bond to the substance. The resulting glucuronide, which has a much higher water solubility than the original substance, is eventually excreted by the kidneys (Wishart et al., 2022). Thus, metabolic liver diseases will affect glucuronidation (Sharma and Nagalli, 2021). Our findings of downregulated 17-alpha-estradiol-3-glucuronide metabolite produced in the liver after glucuronidation of 17-alpha-estradiol by UDP glucosyl transferase are consistent with this hypothesis. Moreover, to our knowledge, 17-alpha-estradiol-3-glucuronide has been identified in the blood of patients with GAL for the first time, which can serve as a potential prognostic biomarker for GAL concerning liver complications.

Furthermore, the metabolite 16 alpha-hydroxy DHEA 3-sulfatediphosphate derived mainly from the fetus and served as a precursor for placental estriol biosynthesis significantly decreased in the GAL group (Schweigmann et al., 2014). 16 alpha-hydroxy DHEA 3-sulfate is a natural human metabolite in pregnant women’s placenta and breast milk (Wishart et al., 2022). However, breastfeeding should be avoidable in babies with GAL since breast milk contains lactose (Berry, 2021). Thus, it may explain the decreased level of 16 alpha-hydroxy DHEA 3-sulfatediphosphate in patients with GAL.

This study had some limitations, such as the few patients with GAL and the need for the patient group to be used for external validation. Nevertheless, using the metabolomics approach, our study is one of the few to reveal specific metabolite changes between GAL and healthy controls. Our findings serve as the initial step for further investigations in greater detail.

## 5 Conclusion

There is currently no biomarker available to predict life-threatening complications in patients with GAL, which are associated with early death among these patients. This study used the HRMS-based metabolomics approach for the first time to gain new insights into the perturbed biochemical pathways in GAL compared to healthy control and to identify potential predictive biomarkers.

A total of 480 endogenous metabolites were identified, and they showed significant dysregulation. These metabolites can provide important insights into the pathophysiological state of GAL disorder.

Two metabolites, ganglioside GT1c and PA (8:0/LTE4), had the highest discrimination between GAL and the healthy group. Moreover, our results showed novel potential biomarkers for GAL, such as 17-alpha-estradiol-3-glucuronide and 16 alpha-hydroxy DHEA 3-sulfatediphosphate.

However, the biomarkers obtained through untargeted metabolomics require additional validation, which may involve the targeted UPLC/HRMS-based method to ensure their accuracy and reliability for clinical use. In addition, further studies are necessary to evaluate these biomarkers’ reproducibility, stability, and performance in large separate cohorts to determine their potential clinical value.

## Data Availability

The data were deposited in a MetaboLight accession number MTBLS6996.

## References

[B1] Banford S. Timson D. J. (2021). The structural and molecular biology of type IV galactosemia. Biochimie 183, 13–17. 10.1016/j.biochi.2020.11.001 33181226

[B2] Banford S. McCorvie T. J. Pey A. L. Timson D. J. (2021). Galactosemia: Towards pharmacological chaperones. J. Pers. Med. 11 (2), 106. 10.3390/jpm11020106 33562227 PMC7914515

[B3] Berry G. T. (2015). “Disorders of galactose metabolism,” in Rosenberg's molecular and genetic basis of neurological and psychiatric disease. Editor Pascual R. N. R. a. J. M. (Elsevier), 615–626.

[B4] Berry G. T. (2021). “Classic galactosemia and clinical variant galactosemia,” in GeneReviews. Editors Margaret D. B. E. Adam P. Mirzaa G. M. Pagon R. A. Wallace S. E. Bean L. J. H. Gripp K. W. (Seattle: University of Washington), 1993–2023. 20301691

[B5] Coman D. J. Murray D. W. Byrne J. C. Rudd P. M. Bagaglia P. M. Doran P. D. (2010). Galactosemia, a single gene disorder with epigenetic consequences. Pediatr. Res. 67 (3), 286–292. 10.1203/PDR.0b013e3181cbd542 19952866

[B6] Dahabiyeh L. A. Malkawi A. K. Wang X. Colak D. Mujamammi A. H. Sabi E. M. (2020). Dexamethasone-induced perturbations in tissue metabolomics revealed by chemical isotope labeling LC-MS analysis. Metabolites 10 (2), 42. 10.3390/metabo10020042 31973046 PMC7074358

[B7] Dewulf J. P. Marie S. Nassogne M.-C. (2021). Disorders of purine biosynthesis metabolism. Mol. Genet. Metab. 136 (3), 190–198. 10.1016/j.ymgme.2021.12.016 34998670

[B8] Gu X. Al Dubayee M. Alshahrani A. Masood A. Benabdelkamel H. Zahra M. (2020). Distinctive metabolomics patterns associated with insulin resistance and type 2 diabetes mellitus. Front. Mol. Biosci. 7, 609806. 10.3389/fmolb.2020.609806 33381523 PMC7768025

[B9] Hermans M. E. van Weeghel M. Vaz F. M. Ferdinandusse S. Hollak C. E. Huidekoper H. H. (2022). Multi‐omics in classical galactosemia: Evidence for the involvement of multiple metabolic pathways. J. Inherit. Metab. Dis. 45 (6), 1094–1105. 10.1002/jimd.12548 36053831

[B10] Isselbacher K. J. Anderson E. P. Kurahashi K. Kalckar H. M. (1956). Congenital galactosemia, a single enzymatic block in galactose metabolism. Science 123 (3198), 635–636. 10.1126/science.123.3198.635 13311516

[B11] Jaber M. A. Benabdelkamel H. Dahabiyeh L. A. Masood A. AlMalki R. H. Musambil M. (2022). The metabolomics approach revealed a distinctive metabolomics pattern associated with hyperthyroidism treatment. Front. Endocrinol. (Lausanne) 13, 1050201. 10.3389/fendo.2022.1050201 36440210 PMC9685425

[B12] Jacob M. Malkawi A. Albast N. Al Bougha S. Lopata A. Dasouki M. (2018). A targeted metabolomics approach for clinical diagnosis of inborn errors of metabolism. Anal. Chim. Acta 1025, 141–153. 10.1016/j.aca.2018.03.058 29801603

[B13] Jacob M. Lopata A. L. Dasouki M. Abdel Rahman A. M. (2019). Metabolomics toward personalized medicine. Mass Spectrom. Rev. 38 (3), 221–238. 10.1002/mas.21548 29073341

[B14] Jacob M. Nimer R. M. Alabdaljabar M. S. Sabi E. M. Al-Ansari M. M. Housien M. (2022). Metabolomics profiling of nephrotic syndrome towards biomarker discovery. Int. J. Mol. Sci. 23 (20), 12614. 10.3390/ijms232012614 36293474 PMC9603939

[B15] Janeckova H. Kalivodova A. Najdekr L. Friedecky D. Hron K. Bruheim P. (2015). Untargeted metabolomic analysis of urine samples in the diagnosis of some inherited metabolic disorders. Biomed. Pap. Med. Fac. Univ. Palacky. Olomouc Czech. Repub. 159 (4), 582–585. 10.5507/bp.2014.048 25482736

[B16] Jans J. J. M. Broeks M. H. Verhoeven-Duif N. M. (2022). Metabolomics in diagnostics of inborn metabolic disorders. Curr. Opin. Syst. Biol. 29, 100409. 10.1016/j.coisb.2021.100409

[B17] Jernigan H. M. Jr Blum P. S. Chakrabarti I. Su Y. Zigler J. S. Jr (2005). Effects of cataractogenesis on the CDP-choline pathway: Increased phospholipid synthesis in lenses from galactosemic rats and 13/N Guinea pigs. Ophthalmic Res. 37 (1), 7–12. 10.1159/000082764 15604593

[B18] Knerr I. Coss K. P. Kratzsch J. Crushell E. Clark A. Doran P. (2015). Effects of temporary low-dose galactose supplements in children aged 5–12 y with classical galactosemia: A pilot study. Pediatr. Res. 78 (3), 272–279. 10.1038/pr.2015.107 26053138

[B19] Kotb M. A. Mansour L. Shamma R. A. (2019). Screening for galactosemia: Is there a place for it? Int. J. Gen. Med. 12, 193–205. 10.2147/IJGM.S180706 31213878 PMC6537461

[B20] Lehotay D. Hall P. Lepage J. Eichhorst J. Etter M. Greenberg C. (2011). LC–MS/MS progress in newborn screening. Clin. Biochem. 44 (1), 21–31. 10.1016/j.clinbiochem.2010.08.007 20709048

[B21] Leloir L. F. (1951). The enzymatic transformation of uridine diphosphate glucose into a galactose derivative. Arch. Biochem. Biophys. 33 (2), 186–190. 10.1016/0003-9861(51)90096-3 14885999

[B22] López de Frutos L. Almeida F. Murillo-Saich J. Conceição V. A. Guma M. Queheberger O. (2022). Serum phospholipid profile changes in gaucher disease and Parkinson’s disease. Int. J. Mol. Sci. 23 (18), 10387. 10.3390/ijms231810387 36142296 PMC9499334

[B23] Masood A. Jacob M. Gu X. Abdel Jabar M. Benabdelkamel H. Nizami I. (2021). Distinctive metabolic profiles between Cystic Fibrosis mutational subclasses and lung function. Metabolomics 17 (1), 4–19. 10.1007/s11306-020-01760-5 33394183

[B24] Ng B. G. Wolfe L. A. Ichikawa M. Markello T. He M. Tifft C. J. (2015). Biallelic mutations in CAD, impair de novo pyrimidine biosynthesis and decrease glycosylation precursors. Hum. Mol. Genet. 24 (11), 3050–3057. 10.1093/hmg/ddv057 25678555 PMC4424951

[B25] Ohlsson A. Guthenberg C. Döbeln U. v. (2011). “Galactosemia screening with low false-positive recall rate: The Swedish experience,” in JIMD reports-case and research reports (Springer), 113–117. 10.1007/8904_2011_59 PMC3509849 23430863

[B26] Pasquali M. Yu C. Coffee B. (2018). Laboratory diagnosis of galactosemia: A technical standard and guideline of the American College of medical genetics and Genomics (ACMG). Genet. Med. 20 (1), 3–11. 10.1038/gim.2017.172 29261178

[B27] Patti G. J. Yanes O. Siuzdak G. (2012). Innovation: Metabolomics: The apogee of the omics trilogy. Nat. Rev. Mol. Cell. Biol. 13 (4), 263–269. 10.1038/nrm3314 22436749 PMC3682684

[B28] Peduto A. Spada M. Alluto A. Dolcetta M. L. Ponzone A. Santer R. (2004). A novel mutation in the GLUT2 gene in a patient with Fanconi-Bickel syndrome detected by neonatal screening for galactosaemia. J. Inherit. Metab. Dis. 27 (2), 279–280. 10.1023/b:boli.0000028841.00833.f4 15243984

[B29] Sahoo T. Thukral A. Agarwal R. Sankar M. J. (2015). Galactosaemia: An unusual cause of chronic bilirubin encephalopathy. BMJ Case Rep. 2015, bcr2014206852. 10.1136/bcr-2014-206852 PMC4307093 25618877

[B30] Schweigmann H. Sánchez-Guijo A. Ugele B. Hartmann K. Hartmann M. F. Bergmann M. (2014). Transport of the placental estriol precursor 16α-hydroxy-dehydroepiandrosterone sulfate (16α-OH-DHEAS) by stably transfected OAT4-SOAT-and NTCP-HEK293 cells. J. Steroid Biochem. Mol. Biol. 143, 259–265. 10.1016/j.jsbmb.2014.03.013 24717977

[B31] Sharma A. Nagalli S. (2021). “Chronic liver disease,” in StatPearls (Florida: StatPearls Publishing). [Internet]. 32119484

[B32] Succoio M. Sacchettini R. Rossi A. Parenti G. Ruoppolo M. (2022). Galactosemia: Biochemistry, molecular genetics, newborn screening, and treatment. Biomolecules 12 (7), 968. 10.3390/biom12070968 35883524 PMC9313126

[B33] Taylor Fischer S. Frederick A. B. Tran V. Li S. Jones D. P. Fridovich-Keil J. L. (2019). Metabolic perturbations in classic galactosemia beyond the Leloir pathway: Insights from an untargeted metabolomic study. J. Inherit. Metab. Dis. 42 (2), 254–263. 10.1002/jimd.12007 30667068 PMC6414239

[B34] Timson D. J. (2016). The molecular basis of galactosemia—past, present and future. Gene 589 (2), 133–141. 10.1016/j.gene.2015.06.077 26143117

[B35] van Weeghel M. Welling L. Treacy E. P. Wanders R. J. Ferdinandusse S. Bosch A. M. (2018). Profiling of intracellular metabolites produced from galactose and its potential for galactosemia research. Orphanet J. Rare Dis. 13 (1), 146–147. 10.1186/s13023-018-0888-1 30143026 PMC6109347

[B36] Veiga‐da‐Cunha M. Van Schaftingen E. Bommer G. T. (2020). Inborn errors of metabolite repair. J. Inherit. Metab. Dis. 43 (1), 14–24. 10.1002/jimd.12187 31691304 PMC7041631

[B37] Vukelic Z. Metelmann W. Müthing J. Kos M. Peter-Katalinic J. (2001). Anencephaly: Structural characterization of gangliosides in defined brain regions. Biol. Chem. 382, 259–274. De Gruyter. 10.1515/BC.2001.033 11308024

[B38] Walter J. H. Fridovich-Keil J. L. (2019). “Galactosemia,” in The online metabolic and molecular bases of inherited disease. Editors Valle D. L. Antonarakis S. Ballabio A. Beaudet A. L. Mitchell G. A. (New York, NY: McGraw-Hill Education), 381.

[B39] Welsink-Karssies M. M. Ferdinandusse S. Geurtsen G. J. Hollak C. E. M. Huidekoper H. H. Janssen M. C. H. (2020). Deep phenotyping classical galactosemia: Clinical outcomes and biochemical markers. Brain Commun. 2 (1), fcaa006. 10.1093/braincomms/fcaa006 32954279 PMC7425409

[B40] Wishart D. S. Guo A. Oler E. Wang F. Anjum A. Peters H. (2022). HMDB 5.0: The human metabolome database for 2022. Nucleic Acids Res. 50 (D1), D622–d631. 10.1093/nar/gkab1062 34986597 PMC8728138

[B41] Worley B. Powers R. (2013). Multivariate analysis in metabolomics. Curr. Metab. 1 (1), 92–107. 10.2174/2213235X11301010092 PMC4465187 26078916

